# Nutritional Patterns and Dietary Imbalances in the Adult Population of Bihor County, Romania (2015 to 2024): A Decade of Nutritional Surveillance

**DOI:** 10.7759/cureus.89286

**Published:** 2025-08-03

**Authors:** Razvan Rahota, Monica Laslau, Dorel Tirt, Diana Rahota, Andreea Camarasan, Ovidiu Pop

**Affiliations:** 1 Department of Morphological Disciplines, University of Oradea, Medicine and Pharmacy Faculty, Oradea, ROU; 2 Department of Psychoneurosciences and Rehabilitation, University of Oradea, Medicine and Pharmacy Faculty, Oradea, ROU

**Keywords:** covid-19 pandemic, increased lipid intake, low intake of fruits, low intake of vegetables, nutritional patterns

## Abstract

Introduction

Dietary intake patterns have a significant impact on overall health, affecting physical well-being through factors such as weight management, metabolic balance, and disease prevention. Additionally, diet affects psychological well-being by influencing mood, cognitive function, and emotional stability.

The study evaluates the dietary habits of the adult population in Bihor County over a 10-year period, structured into three major intervals: the pre-pandemic years (2015-2019), the COVID-19 pandemic period (March 2020-March 2022), and the post-pandemic years (2023-2024).

Materials and methods

All data were collected over a time period between January 2015 and December 31, 2024, from Bihor County, Romania. Statistical analysis was performed using the Jamovi (Version 2.2.5.0) [computer software]. One-way analysis of variance (ANOVA), Welch's ANOVA, Fisher's test, and Tukey's post-hoc test were used to compare the results.

Results

Based on 24-hour recall surveys and standardized nutritional analysis, the study reveals persistent imbalances in macronutrient intake and food group distribution. Excess lipid intake, reduced carbohydrate consumption, and insufficient intake of fish, fruits, and vegetables were consistent across all periods, with significant exacerbation during the pandemic.

Conclusions

The findings underscore the need for regionalized interventions to promote sustainable and balanced dietary practices. The COVID-19 pandemic acted as an aggravating factor, further worsening dietary quality and caloric balance. Although a slight improvement has been observed in the post-pandemic period, the persistence of unhealthy eating habits, especially excessive fat consumption and insufficient intake of nutrient-rich foods, remains a significant concern.

## Introduction

Human dietary behavior is not only a survival mechanism but also a lifestyle expression with profound implications for health and well-being. Despite increasing awareness, prevalent errors in daily nutritional practices persist, often based on cultural traditions that, while fostering identity and continuity, lack the necessary diversity and balance for optimal health [1,2]. 

In Bihor County, Romania, the interaction of dietary customs, nutritional deficiencies, and overconsumption of specific food groups presents a complex public health challenge. Both rural and urban populations face significant nutritional inadequacies, particularly in energy and macronutrient distribution. Cultural dietary models and socioeconomic status further modulate these patterns, influencing food choices and health outcomes [1,2].

Previous studies indicate consistent caloric and nutritional deficits among Bihor County residents, especially regarding vegetable fats and a surplus of animal proteins. While micronutrient intake generally aligns with recommended levels, the temporal distribution of calorie intake across meals is suboptimal; breakfast and lunch align more closely with dietary norms, while other meals fall short. Rural populations consume more saturated fats and fewer dietary fibers and carbohydrates than their urban counterparts [3,4].

The region’s diet is characterized by high consumption of meat, dairy products, and refined cereals, and low intake of milk, fresh produce, and fish [5]. Such preferences reflect a nutritionally unsustainable model, often deficient in dietary fiber due to low consumption of whole grains, fruits, and vegetables, factors that are associated with digestive disorders and chronic illnesses [6]. Romanian culinary traditions, while culturally significant, do not always align with sustainable dietary practices, highlighting the need for a transition toward more diverse, plant-based alternatives [7].

Socioeconomic factors, including income and education, also influence dietary patterns [8]. Lower-income households typically depend on inexpensive, high-calorie foods like bread and grains, while higher-income households have greater access to fresh produce and lean proteins. 

The COVID-19 pandemic disrupted dietary habits further, increasing intake of fats and carbohydrates while reducing protein intake. These shifts have had long-term consequences, including heightened risk for obesity and diet-related diseases, and potentially even sex-specific acceleration of aging due to vitamin deficiencies [9]. 

Elevated consumption of proteins and saturated fats correlates with rising overweight and obesity rates in Romania, including among children. National dietary interventions are imperative to address these imbalances and promote healthier eating habits [10].

Aim and scope

The study aims to evaluate long-term trends in dietary intake among the adult population of Bihor County, Romania, over a 10-year period (2015-2024). The primary objectives are to identify changes in caloric and macronutrient intake across three key periods (pre-pandemic, pandemic, and post-pandemic), to assess the prevalence of dietary imbalances in the studied population, and to provide data-driven insights for public health strategies aimed at guiding regional interventions, informing educational initiatives, and preventing diet-related diseases.

## Materials and methods

Study design

The current study is a retrospective, observational one conducted over a 10-year period, from January 2015 to December 2024, in Bihor County, Romania. The research aimed to assess changes and trends in dietary intake patterns among adults aged over 20, with data stratified into three major periods based on the COVID-19 pandemic timeline: pre-pandemic (2015-2019), pandemic (March 2020-March 2022), and post-pandemic (2023-2024).

The study was conducted through collaboration between academic and clinical institutions in Oradea, including the University of Oradea, the Public Health Department, and the Bihor County Clinical Hospital.

Study population and sampling

A cohort of 474 adult individuals, 258 women and 216 men, were enrolled in the present study across the 10-year period. The participants were selected to represent both urban (n = 267) and rural (n = 207) populations of Bihor County. Although each year’s sample was not longitudinally followed, the aggregate data were used to reflect trends across the time intervals.

Inclusion Criteria

Individuals over 20 years of age at the time of participation, with permanent residency in Bihor County for at least the previous 12 months, were included. Only the participants without acute illnesses or chronic conditions requiring therapeutic diets, such as diabetes or renal failure, were considered. Additionally, participation in the study required informed consent, including the agreement to complete a 24-hour dietary recall questionnaire. The 24-hour dietary recall questionnaire was administered in face-to-face interviews by trained personnel. Participants were asked to report all foods and beverages consumed during the previous day, including the time of consumption and preparation method. Food items were classified by major food groups and subgroups (e.g., red meat: pork, lamb, beef; cereals: white flour, whole grains, cereal derivatives). Additional information collected included the number of meals per day and the estimated quantity of each item. All responses were reviewed for completeness and internal consistency prior to statistical analysis. The same standardized procedure was maintained throughout the 10-year data collection period.

Exclusion Criteria

Individuals with incomplete or inconsistent data from the dietary recall, as well as pregnant or lactating women, due to their altered nutritional requirements, were excluded from the study. Participants following medically prescribed diets or restrictive regimens were also excluded, as these patterns did not reflect typical dietary habits in the general population.

The number of individuals included in the sample each year is presented in Table 1.

**Table 1:** Number of individuals included in the sample by year

Year	2015	2016	2017	2018	2019	2020	2021	2022	2023	2024	Total
Number of individuals	50	60	55	51	51	26	30	50	51	50	474

Macronutrient intake, caloric values, and food group consumption were analyzed per period, and results were expressed as average daily intake and percentage deviation from recommended norms.

Statistical analysis

All the data were initially introduced in Microsoft Excel (Redmond, USA) spreadsheets. Jamovi (Version 2.2.5.0) [computer software] was used for all statistical analyses. For every analysis, the threshold for statistical significance was set at p < 0.05 [11].

Data were grouped into three time intervals (pre-pandemic, pandemic, and post-pandemic) for comparison. The primary outcome measures for statistical comparison were: total energy intake (kcal/day), total fat intake (g/day), total protein intake (g/day), and total carbohydrate intake (g/day).

A one-way analysis of variance (ANOVA) was used to assess variations in dietary intake variables between the three study periods (pre-pandemic, pandemic, and post-pandemic). Total energy intake (kcal), total fat intake (g), total protein intake (g), and total carbohydrate intake (g) were the dependent variables under analysis. The study period, which was treated as a three-level categorical factor, was the independent variable.

The assumptions of normality and homogeneity of variances were evaluated before the ANOVA was conducted. Welch's ANOVA correction was used to account for unequal variances in situations where the assumption of homogeneity of variances was broken.

After ANOVA identified significant overall differences, Tukey's Honestly Significant Difference (HSD) test was used for post-hoc multiple comparisons to identify which particular pairs of study periods had statistically significant differences in mean dietary intake.

Ethical considerations

Data collection followed ethical standards for human research. The Research Ethical Approval for the study was granted by the Research Ethics Committee Decision of the University of Oradea (approval number CEFMF/33 from 11.01.2022), and written consent was obtained from all participants.

## Results

Among the studied population, more than half of the individuals included in the study were from urban areas. Other demographic information is presented in Table 2. 

**Table 2:** Demographic characteristics table of the studied population

Category	Value (Number)
Total Participants	474
Area of Origin	
Urban Residents	267
Rural Residents	207
Sex	
Women	258
Men	216
Age	
Age limits (years)	20–87
Mean Age	49.9
Median Age	49.5
Standard Deviation (Age)	16.2

Table 3 presents the results of our study regarding the intake of major food groups, compared with the dietary reference values proposed by the EAT-Lancet Commission. The consumption of fresh red meat (76.32 g/day), poultry (76.39 g/day), and sugary products (78.2 g/day) substantially exceeded the recommended levels, while the intake of fish (3.2 g/day), vegetables (162.57 g/day), fruits (124.56 g/day), and dairy products (103.46 g/day) remained well below the suggested targets. The average number of meals per day was 3.63.

**Table 3:** Observed dietary intake versus EAT–Lancet recommendations g: grams

Food Item	Dietary Intake Observed Among the Studied Population (2015–2024)	EAT–Lancet Recommended Intake [12]
Fresh red meat	76.32 g/day	~14 g/day (0–28 g/day)
Poultry meat	76.39 g/day	~ 29 g/day
Processed meat	25.85 g/day	~14 g/day (0–28 g/day) Included in red meat limit
Fish	3.2 g/day	~28 g/day
Vegetables	162.57 g/day	~300 g/day
Fresh fruits	124.56 g/day	~200 g/day
Bread and cereal derivatives	277.24 g/day	~232 g/day
Milk and other derivatives	103.46 g/day	~250 g/day
Sugary products	78.2 g/day	<31 g/day
Potatoes	43.22 g/day	~ 50 g/day (0-100 g/day)

**Figure 1:**
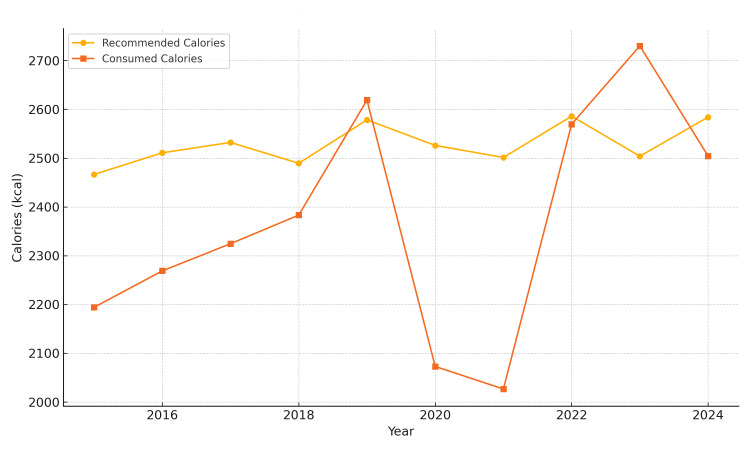
Average daily caloric intake in Bihor County (2015-2024) The image is created by the author.

Throughout the study period, daily caloric intake exhibited mild to moderate deficits, particularly during the pandemic years of 2020 and 2021, which showed the greatest reductions (-17.94% and -18.98%, respectively). By contrast, only two years, 2019 and 2023, recorded surplus intakes (Figure 1).

**Figure 2:**
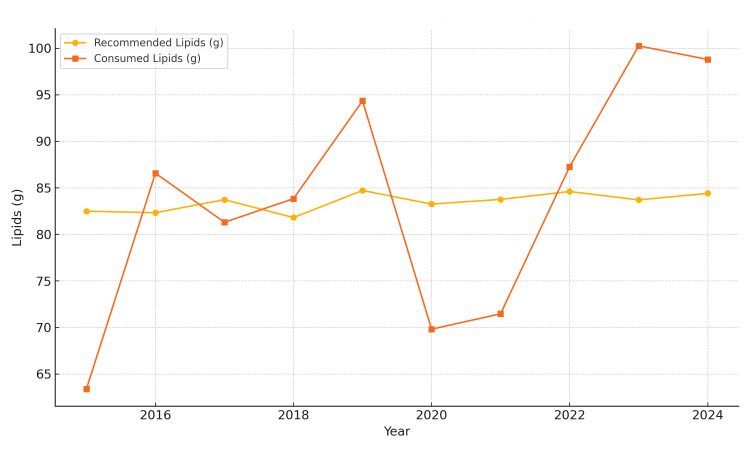
Average daily lipid intake in Bihor County (2015-2024) The image is created by the author.

Lipid consumption (Figure 2) revealed significant excesses in six of the ten years, with the highest deviations noted in 2023 (+19.76%) and 2024 (+17.05%).

**Figure 3:**
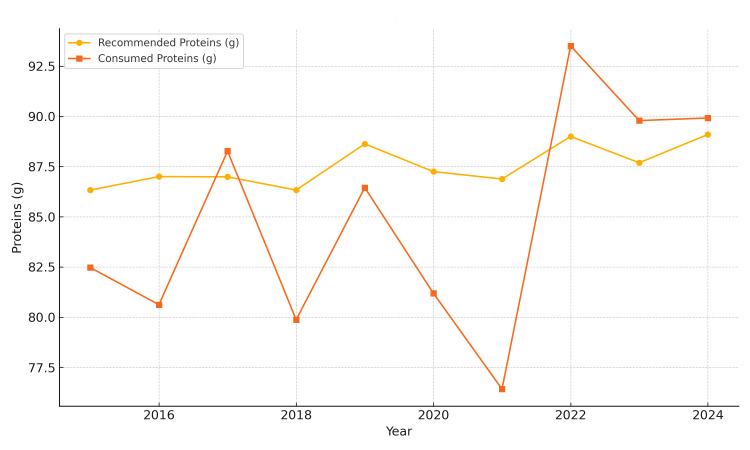
Average daily protein intake in Bihor County (2015-2024) The image is created by the author.

**Figure 4:**
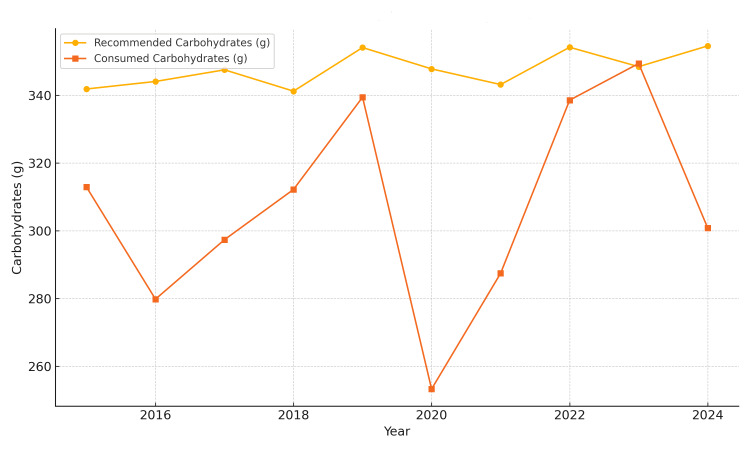
Average daily carbohydrate intake in Bihor County (2015-2024) The image is created by the author.

Protein intake was relatively stable, with only minor deviations (Figure 3), but carbohydrate intake was persistently below recommended levels, reaching a peak deficit of -27.18% in 2020 (Figure 4).

To better understand the influence of broader societal and health-related shifts, the analysis of dietary patterns was structured as follows:

Pre-pandemic (2015-2019): Average caloric intake remained consistently below recommended values. Notable deficiencies in carbohydrate intake (−20%) were observed, along with moderate lipid excess (+10-15%). Protein intake was near optimal, with an average of approximately 36.5 g/day from meat (based on a total meat intake of 156.9 g/day), covering approximately 80-85% of daily protein needs for women and 60-65% for men. Fruit and vegetable intake fell below 60% of the recommended levels (mean: 116.21 g/day and 148.5 g/day, respectively), while fish intake (1.63 g/day) remained critically low.

COVID-19 pandemic (March 2020-March 2022): Marked decline in overall caloric intake (−18% in 2020). Lipid intake increased sharply (up to +20%), and carbohydrate intake fell to −25-30% of the recommended daily intake (RDI). Sugary product consumption peaked in 2021, with an average intake of 106.86 g/day. Meal frequency declined to 2.9 meals per week, particularly affecting breakfast. Fish intake remained extremely low (1.63 g/day), and fresh produce consumption consistently failed to meet 60% of dietary recommendations.

**Figure 5:**
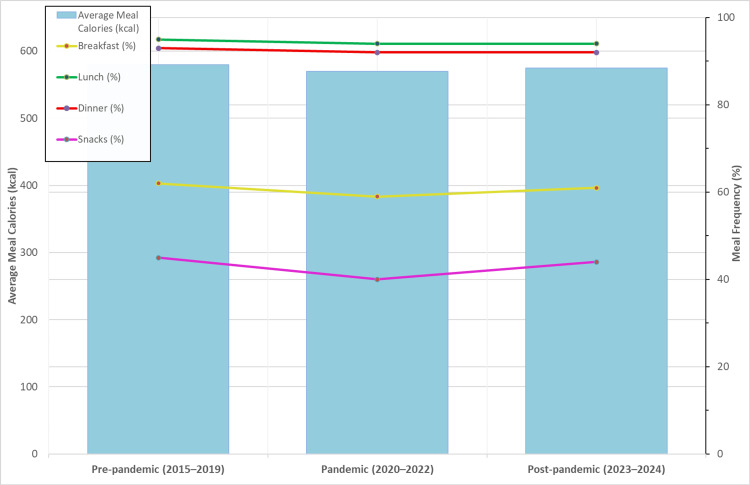
Average meal calories and frequency across periods (2015–2024) The image is created by the author.

Post-pandemic (2023-2024): Partial recovery in caloric intake, yet levels remained insufficient for some demographic groups. Lipid consumption remained high (+17.1%), and carbohydrate intake improved marginally. Protein intake returned to earlier levels, still dominated by meat. Nutritional quality of cereals and frequency of breakfast consumption remained suboptimal (Figure 5).

Table 4 summarizes the statistically significant differences observed for total energy intake and total fat intake, while protein and carbohydrate intake did not vary significantly (p > 0.05 for both). Post-hoc analysis using Tukey’s HSD test revealed that mean energy intake in the post-pandemic period was significantly higher than in the pre-pandemic period. The post-pandemic period also showed noticeably higher fat intake compared to both the pre-pandemic and pandemic periods. In contrast, protein and carbohydrate consumption remained relatively stable across all three time intervals.

**Table 4:** Statistical analysis of nutrient intake by period

Dependent Variable	Welch's F (df=2,201)	p-value	Post-hoc (Tukey HSD)
Energy Intake (kcal)	3.353	0.037	Post-pandemic > Pre-pandemic (−268 kcal, p = 0.026)
Fat Intake (g)	4.664	0.011	Post-pandemic > Pre-pandemic (−17.2 g, p = 0.007); post-pandemic > Pandemic (−20.4 g, p = 0.007)
Protein Intake (g)	Not significant	> 0.05	No significant difference
Carbohydrate Intake (g)	Not significant	> 0.05	No significant difference

These findings indicate that, following the COVID-19 epidemic, the energy and fat intake of the study population notably changed; this could be because of psychological variables, dietary practices, or lifestyle choices.

## Discussion

The current study of dietary habits in Bihor County highlights a generally calorically deficient intake, particularly during the pandemic, with consistent imbalances in macronutrient consumption. While protein and lipid levels were closer to recommended values, carbohydrate intake was insufficient. Qualitative assessment shows persistent shortages in vegetable fats and excesses in animal protein intake. 

Comparing our results with the dietary recommendations proposed by the EAT-Lancet Commission, we observed that in Bihor County, the most frequently overconsumed food items included meat (particularly pork and poultry), processed meats, cereal derivatives, and sugary products. In contrast, fish, vegetables, potatoes, fresh fruits, and milk were consistently under-consumed. Bread intake also remained below the recommended levels throughout the study period [12]. Although the average number of meals per day was within acceptable limits, the distribution and structure of meals contributed to these dietary imbalances. Late and heavy evening meals, along with the frequent lack of breakfast and snacks, may further exacerbate metabolic risks. 

The unifactorial analysis of variance revealed notable variations in terms of caloric intake and total fat intake across the three periods (pre-pandemic, pandemic, and post-pandemic) within the investigated group. The findings show a significant increase in energy and lipid intake in the post-pandemic period as compared to the pre-pandemic period, implying a possible change in the eating behavior of the population once the limitations caused by the COVID-19 epidemic relax. 

Literature also helps to support the variations among the three periods. Adults often kept a rather consistent eating pattern in the pre-pandemic era, marked by particular macronutrient distribution and calorie consumption. Research shows an average daily calorie consumption of between 2,000 and 2,500 kcal, much influenced by lifestyle choices, socioeconomic level, and cultural preferences [13]. Especially, this era was characterized by a balance in the intake of several nutrients, often in line with dietary guidelines provided by medical institutions [14]. Many people have experienced more stress, economic uncertainty, and lifestyle disruptions; thus, the worldwide epidemic has triggered notable changes in eating habits. Evidence shows a rise in home cooking and a change toward eating snack patterns, which enables us to explain weight gain and changes in nutrient intake. American adults reported higher consumption of processed foods and sugar-sweetened beverages, together with an increase in caloric intake, particularly among some demographic cohorts [15]. Several studies developed in Iran (Moursi et al., Hajipoor et al.) noted that dietary processes differ depending on socioeconomic level; low-income homes see more increases in caloric intake because of limited food options and stockpiling behavior during lockdowns [16,17]. Other research points to a lower calorie consumption during the epidemic. Particularly women, medical students ate fewer carbohydrates, which resulted in fewer calories consumed overall [18]. In rural China, caloric consumption decreased by 1.30% for every 100 confirmed cases, a finding that reflects broader socioeconomic and behavioral changes triggered by the pandemic [19]. Other studies, such as those involving the Korean adult population, in which caloric intake stayed constant with changes in eating habits, including increased meat consumption and reduced consumption of fruits and vegetables, do not show notable variations in energy intake during the epidemic [20].

Different eating patterns have surfaced as the globe entered the post-pandemic stage. In a study on post-COVID eating behavior changes, some participants said they returned to pre-pandemic practices of high-calorie snacking and fast food, while others expressed an enhanced dedication to better eating practices. For instance, Sahudin et al. recorded a daily sugar intake rise in Malaysia following the pandemic, thus reflecting a continuous influence of the epidemic on eating behavior [21]. Energy intake dropped during quarantine, according to a longitudinal study conducted in France, but it returned to baseline after one year [22]. 

Furthermore, studies spanning several cohorts revealed that some adults went back to former calorie intake levels while others still struggled with weight control and nutritional balance. Variations in the desire to preserve healthier habits modified during the pandemic suggest the need for continuous public health campaigns to support dietary changes in the future [16,23]. The relationship between nutrition, dietary habits, and the management of obesity and chronic diseases is evident. Utilizing technology and therapeutic innovations, in conjunction with behavioral strategies, facilitates the prevention and management of diseases associated with nutritional deficiencies and unhealthy eating behaviors [24,25]. 

To address these challenges, we recommend the following strategies: A) Implementation of dedicated nutritional education sessions in kindergartens and schools [26]. B) Organization of adult education programs on healthy eating and culinary practices. C) Public education initiatives on food labeling and nutritional content [27]. D) Increased efforts in public awareness campaigns to promote balanced dietary behaviors, especially targeting children, adolescents, and young adults forming families, to prevent the onset of nutrition-related metabolic diseases and their complications [28,29].

Limitations of the study

The study has several limitations that should be acknowledged. First, a major limitation is represented by the sample size; although the study covers a decade, the average number of participants per year was 50, which may limit the statistical power of the findings. Additionally, the use of single 24-hour dietary recalls may not accurately reflect habitual intake. Another major limitation of the study is its cross-sectional design, which does not follow the same individuals longitudinally, thereby limiting the ability to assess causal changes in dietary behavior over time. Individual factors such as age, sex, body mass index, and habitual factors like monthly income and levels of physical activity were not assessed, which limits the ability to interpret the dietary data in relation to health status, socioeconomic context, and lifestyle influences. Comorbidities were not followed, as they do not reflect the dietary patterns of the general population.

## Conclusions

The research reveals persistent nutritional imbalances among the adult population of Bihor County, with only modest improvement over a 10-year span. The COVID-19 pandemic exacerbated the existing dietary inadequacies, particularly by increasing fat consumption and reducing overall energy intake. Although caloric intake showed partial recovery in the post-pandemic period, fat, especially saturated fat, remained elevated, while carbohydrate intake continued to fall short of recommended levels. The macronutrient profile consistently reflected excessive fat intake, slightly elevated protein (mostly animal-derived), and insufficient carbohydrate consumption.

These findings confirm significant changes in energy and lipid intake across the three time periods, particularly in the post-pandemic years. The data suggests the urgent need for multifaceted nutritional interventions tailored to regional contexts. These should include educational programs at community and institutional levels, policy measures to enhance food diversity and affordability, and efforts to promote sustainable dietary models aligned with flexitarian or Mediterranean patterns. Without such systemic changes, the long-term health burden of diet-related diseases is likely to escalate.
